# Diarrhea-Causing Bacteria and Their Antibiotic Resistance Patterns Among Diarrhea Patients From Ghana

**DOI:** 10.3389/fmicb.2022.894319

**Published:** 2022-05-19

**Authors:** Theophilus Afum, Diana Asema Asandem, Prince Asare, Adwoa Asante-Poku, Gloria Ivy Mensah, Abdul Basit Musah, David Opare, Kiyosi Taniguchi, Nuhu Muniru Guinko, Thelma Aphour, Doris Arhin, Koichi Ishikawa, Tetsuro Matano, Taketoshi Mizutani, Franklin Asiedu-Bekoe, Hiroshi Kiyono, Abraham Kwabena Anang, Kwadwo Ansah Koram, Dorothy Yeboah-Manu

**Affiliations:** ^1^College of Health Sciences, Noguchi Memorial Institute for Medical Research, University of Ghana, Accra, Ghana; ^2^Ghana Health Service, Accra, Ghana; ^3^The Institute of Medical Science, The University of Tokyo, Tokyo, Japan; ^4^Graduate School of Medicine, Institute for Global Prominent Research, Chiba University, Chiba, Japan; ^5^Joint Research Center for Human Retrovirus Infection, Kumamoto University, Kumamoto, Japan; ^6^Department of Medicine, CU-UCSD Center for Mucosal Immunology, Allergy and Vaccines (cMAV), University of California San Diego, San Diego, CA, United States

**Keywords:** diarrhea, bacteria, resistance, antimicrobial, susceptibility

## Abstract

Diarrheal disease remains a major global health problem particularly in children under 5 years and the emergence of antibiotic-resistant strains of causative pathogens could slow control efforts, particularly in settings where treatment options are limited. This surveillance study conducted in Ghana aimed to determine the prevalence and antimicrobial susceptibility profile of diarrhea-causing bacteria. This was a cross-sectional study carried out in five health facilities in the Ga West Municipality of Ghana between 2017 and 2021. Diarrheic stool samples from patients were collected and cultured on standard differential/selective media and isolates identified by standard biochemical tests, MALDI-TOF assay, and serological analysis. The antibiogram was determined using Kirby-Bauer disk diffusion and Microscan autoScan4 MIC panels which were used for extended-spectrum beta-lactamase (ESBL) detection. Bacteria were isolated from 97.5% (772/792) of stool samples, and 167 of the isolates were diarrheagenic and met our inclusion criteria for antimicrobial resistance (AMR) analysis. These included *Escherichia coli* (49.1%, 82/167), *Salmonella* species (23.9%, 40/167), *Vibrio* species (16.8%, 28/167), and *Shigella* species (10.2%, 17/167). Among 24 *Vibrio* species, we observed resistances to cefotaxime (21/24, 87.5%), ceftriaxone (20/24, 83.3%), and ciprofloxacin (6/24, 25%), including four multi-drug resistant isolates. All 13 *Vibrio parahaemolyticus* isolates were resistant to cefazolin. All 17 *Shigella* isolates were resistant to tetracycline with resistance to shigellosis drugs such as norfloxacin and ciprofloxacin. *Salmonella* isolates were highly susceptible to norfloxacin (40/40, 100%) and tetracycline (12/34, 35%). Two ESBL-producing *E. coli* were also identified with marked susceptibility to gentamicin (66/72, 91.7%) and amikacin (57/72, 79.2%) prescribed in the treatment of *E. coli* infections. This study showed the different bacteria implicated in diarrhea cases in Ghana and the need for differential diagnoses for better treatment outcomes. *Escherichia coli*, *Shigella*, *Salmonella*, and *Vibrio* have all been implicated in diarrhea cases in Ghana. The highest prevalence was *E. coli* and *Salmonella* with *Shigella* the least prevalent. Resistance to commonly used drugs found in these isolates may render bacteria infection treatment in the near future nearly impossible. Routine antimicrobial susceptibility testing, effective monitoring, and nationwide surveillance of AMR pathogens should be implemented to curb the increase of antimicrobial resistance in Ghana.

## Introduction

Diarrhea disease, although preventable and highly treatable is the second leading cause of death among children under the age of five globally, killing about 525,000 children annually (World Health Organization, 2017). Diarrhea as defined by WHO is the excessive and frequent evacuation of watery stools usually indicating gastrointestinal disease or disorder of 3–7 days duration (World Health Organization, 2017). The etiology of diarrheal diseases mainly by a wide variety of viral, bacterial, and parasitic pathogens is variable depending on a range of conditions including but not limited to geographic and climate conditions, host factors, and socioeconomic situations (Hodges and Gill, 2010). Viral pathogens such as norovirus and rotavirus infection are known to be a leading cause of diarrhea among children under 2 years with the latter causing the highest fatality among children (Tate et al., 2016). Parasites such as *Entamoeba histolytica*, *Giardia lamblia*, and *Cryptosporidium parvum* have also been recorded to cause morbid diarrhea episodes (Nkrumah and Nguah, 2011; Gilchrist et al., 2016; Yang et al., 2021).

Among the bacterial causes, *Escherichia coli* is the most common pathogen for childhood diarrhea in developing countries and an emerging antimicrobial-resistant entero-pathogen in developed countries (Zhou et al., 2018). Bacteria pathogens causing diarrhea-associated diseases have however been somewhat limited to *Vibrio cholerae* with some strains such as Ogawa capable of causing epidemics when not well managed (Danso et al., 2020). In Sub-Saharan Africa, diarrhea is known to cause high fatality among children in rural and low-income communities as compared to the Americas and Europe. This is mainly because of unsanitary conditions such as defecation into rivers and streams which double as drinking water for livestock and humans, lack of potable drinking water, and improper handling of household food can lead to increased diarrhea cases (Larbi et al., 2021; Takyi et al., 2021).

In Ghana, cholera outbreaks and epidemics were recorded in 1970, 2012, 2014, and 2015. The 2014–2015 epidemic affected all 10 regions of the country accounting for a total of 28,922 cases including 243 deaths (Government of Ghana, 2015; Noora et al., 2017) with observed clustering of isolates in some areas of southern Ghana (Danso et al., 2020). However, it has been established that diarrhea can be caused by different types of bacteria other than *V. cholerae* including *Campylobacter* spp., *Salmonella* spp., *Shigella* spp., and *pathogenic Escherichia coli* O15:H7 (although unresolved), and can be equally fatal when left untreated (William et al., 2020; Wang et al., 2021).

Infections with these bacteria are often easily treated with Oral Rehydration Solutions (ORS) and zinc as recommended by WHO; however, some patients require antimicrobial therapy in cases of bloody, severe, or persistent diarrhea (World Health Organization, 2005). With increasing reports of high antibiotic resistance among enterobacteria in Ghana, the treatment of diarrhea caused by bacteria will be a challenge (Kunadu et al., 2018; Obeng-Nkrumah et al., 2019). Reports of over 40% of non-bacterial diarrhea in children being treated with antibiotics, easy accessibility to antibiotics through unapproved means in Ghana, and wrongful prescription of drugs are of grave concern (Arhinful, 2009; Ahiabu et al., 2016). There are some studies implicating bacteria in diarrhea cases in Ghana (Akuffo et al., 2017; Mizutani et al., 2021), with a prevalence of rate of 3% and high resistance to commonly abused drugs such as tetracycline (Newman et al., 2011; Akuffo et al., 2017). This study aimed to isolate bacterial organisms associated with diarrhea in patients reporting to five health facilities in Ga West, Accra-Ghana, and determine the antimicrobial susceptibility profile of the isolates.

## Materials and Methods

### Ethical Considerations

This cross-sectional surveillance study conducted from August 2017 to May 2021 was approved by the Institutional Review Board (IRB) of Noguchi Memorial Institute for Medical Research (NMIMR; approval number: 096/16-1; dated May 3, 2017). It was exempted from additional approval by the Ghana Health Service Ethics Committee which considered the procedures as compliant with routine service for patients care and surveillance.

### Study Area

**Figure 1:**
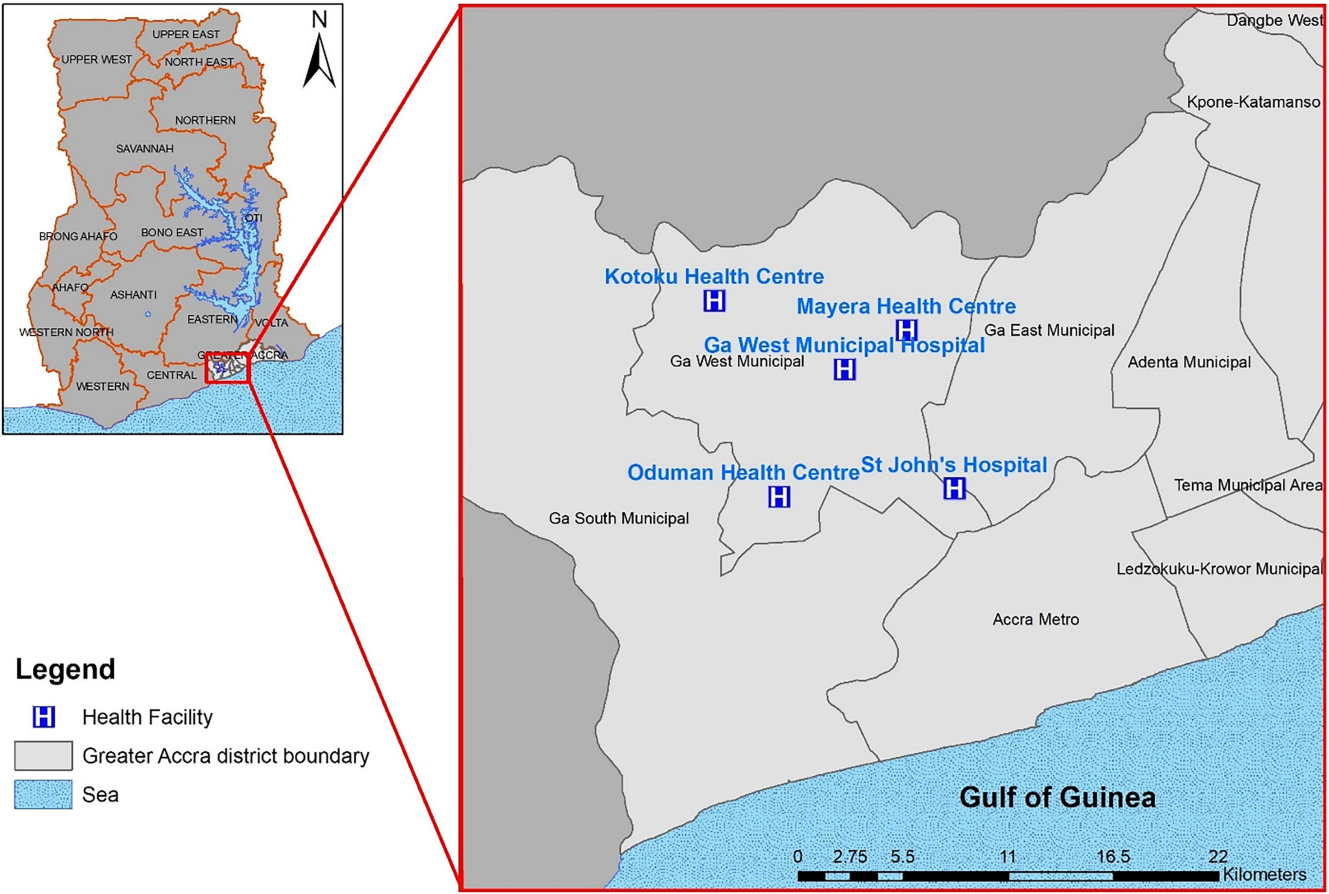
A map showing the geographical locations of the participating health facilities.

The study was conducted in the Ga West Municipality which lies between 5°35′North, 5°29′North, and longitude 0°10′West and 0°24′West. It occupies a total surface area of 299.578 square kilometers with about 412 communities. Five health facilities including a private facility within the municipality were chosen as surveillance sites (Figure 1). The main occupation or industry of inhabitants is trading and sales work of which females dominate. Public toilets and pit latrines are the main toilet facilities in the Municipality (Ghana Statistical Service, 2013).

### Participants’ Data and Sample Collection

Individuals passing watery or loose stools three or more times within 24 h with/without mucus and or blood, vomiting, fever, and abdominal pain were eligible for this study.

Samples were taken between August 2017 and May 2021. Before stool sample collection into a container, patients’ clinical and demographic data including age, gender, height, weight, food, and water taken 3 days before the onset of diarrhea, temperature, number of stools per day, antibiotics taken, places visited among others were recorded using a structured questionnaire.

All collected stool samples were screened for *V. cholerae* using a rapid diagnostic test kit (Abbott Bioline, United States) at the various health facilities before transportation to the NMIMR laboratory for analysis. With the aid of a swab stick, a portion of the stool was inoculated into Cary Blair transport medium on-site and transported to NMIMR in a cold chain for culture and antimicrobial susceptibility testing.

### Bacteria Isolation and Characterization

Each sample was inoculated on four media: MacConkey agar (ThermoScientific, United States) for isolation of non-lactose fermenters and *E. coli*, Thiosulphate Citrate Bile Salt agar, TCBS (Park Scientific, United Kingdom) for isolation of *Vibrio* spp., Alkaline Peptone Water, APW (Himedia, India), an enrichment medium for isolation of *Vibrio* spp. and Selenite Faecal Broth, SFB (Himedia, India) an enrichment medium for isolation of *Salmonella* and *Shigella* spp. and then incubated aerobically at 37°C for 18–24 h for isolation of enteric bacteria (Bolinches et al., 1988; Akuffo et al., 2017). A loopful of SFB and APW broth cultures were sub-cultured onto *Salmonella Shigella* Agar (SS) and TCBS respectively and incubated as previously. Suspected colonies of *Salmonella* spp., *Shigella* spp., *E. coli*, and *Vibrio* spp. were purified and identified by colonial characteristics, Gram staining reaction (Park Scientific, United Kingdom), standard biochemical methods-Analytical Profile Index (Biomerieux, United States), and Microscan Autoscan4 (Beckman Coulter, United States), and Matrix-Assisted Laser Desorption/Ionization-Time of Flight, MALDI-TOF (Bruker, United States). *Shigella*, *E. coli*, *Salmonella*, and *Vibrio cholerae* isolates were antigenically characterized using serological kits from Denka Seiken, Japan (Nath et al., 2013; Pun, 2014).

### Antimicrobial Susceptibility Testing

Only isolates that were regarded as diarrheagenic strains were included in our analysis for antimicrobial susceptibility testing. Susceptibility to antimicrobial agents was done by Kirby Bauer disk diffusion technique per Clinical and Laboratory Standard Institute (CLSI) guidelines (CLSI, 2020). Isolates were tested against ceftriaxone (30 μg), norfloxacin (5 μg), cefotaxime (30 μg), amikacin (30 μg), gentamycin (30 μg), clotrimazole (50 μg), nalidixic acid (30 μg), tetracycline (30 μg), ceftazidime (30 μg), ciprofloxacin (5 μg), nitrofurantoin (300 μg), and cefazolin (30 μg; BD BBL, United Kingdom). *Escherichia coli* ATTC 25922 reference strain was used as a control. MicroScan autoScan4 MIC panels-Gram-Negative combo type 66 and Gram-Positive PB 44 (Beckman Coulter, United States) were also used to determine the susceptibility profiles of some isolates. Zones of Inhibition were measured on Mueller Hinton Agar plates in Kirby Bauer method and MIC used in MicroScan autoscan4. Resistance, intermediate susceptibility, and susceptibility were defined according to the CLSI breakpoints (CLSI, 2020).

### ESBL Characterization/Multidrug Resistance

All extended spectrum-beta-lactamase (ESBLs) in this study were identified using a Gram-negative Combo type 66 panel on a MicroScan autoScan-4. This panel detects MIC of drugs and also virulent markers such as ESBL, and carbapenems. Multidrug resistance was defined as isolates being resistant to more than three antibiotic drug classes (Magiorakos et al., 2012).

### Data Management and Analysis

Participants’ clinical, demographic, and laboratory data were entered into Epi Info version 2.0 Software (Centers for Disease Control and Prevention, CDC, Atlanta, GA, United States) and analyzed using Stata v14.2 (Stata Corporation, College Station, TX, United States). Descriptive statistics were carried out for both categorical and numerical variables. Cross-tabulations were further employed to explore the relationship between the different outcomes and selected variables using Chi-square and student *t*-test where applicable. Where appropriate, the Fisher’s exact or the chi-square tests were used to assess statistical significance. A *p*-value of less than 0.05 at a 95% confidence level was considered significant. The ArcMap tool employed in ArcGIS (Economic and Social Research Institute, version 10.1; ESRI, 2010) was used for constructing maps.

## Results

### Demographic and Clinical Characterization of Cases

**Figure 2:**
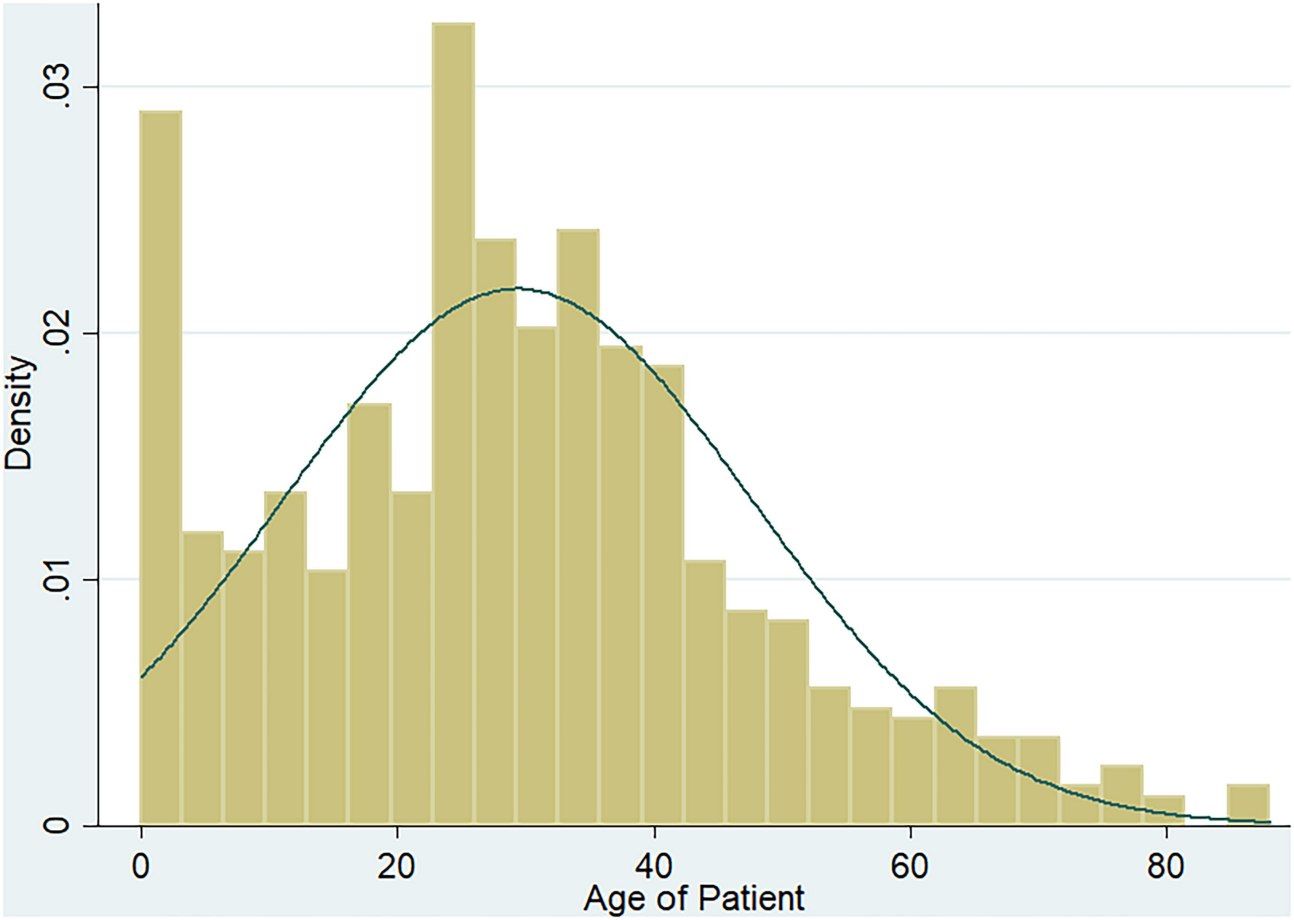
Age distribution of participants.

Seven hundred and ninety-two diarrheic stool samples were collected from the health facilities; 297 (37.5%) males and 495 (62.5%) females. The ages of participants ranged between 5 months and 88 years with the majority (589/792, 74.3%) being adults (>17 years) and a mean age of 29.3 years (Figure 2).

Clinical records indicated that 124 (15.7%) patients had taken antibiotics, 85 (10.7%) antimalarials, 40 (5.0%) anthelminthic, 37 (4.7%) both antibiotics and antimalarials, 17 (2.1%) all three drugs, and 497 (62.8%) had not taken any drug before visiting the health facilities. The highest temperature recorded among participants was 39.8°C and the lowest 30.1°C with a mean temperature of 36.3°C. Symptoms such as fever (347, 36.8%) vomiting (374, 47.2%), weakness (529, 66.8%), and muscle cramps (297, 37.5%) were recorded among the patients (Table 1).

**Table 1:** Symptoms presented by participants.

Vomiting	Frequency *N* (%)
Present	374 (47.2)
Absent	388 (49.0)
Not specified	30 (3.8)
**Muscular cramps**	**Frequency**
Present	297 (37.5)
Absent	408 (51.5)
Not specified	87 (11.0)
**Weakness**	**Frequency**
Present	529 (66.8)
Absent	231 (29.2)
Not specified	32 (4.0)
**Fever**	**Frequency**
High grade	110 (13.9)
Low grade	237 (23.9)
Absent	350 (44.2)
Not specified	95 (12.0)

### Distribution of Diarrheic Bacteria Characterized

Bacterial isolates were obtained from 772/792 stool samples collected; 21.6% (167/772) were diarrheagenic bacteria including *E. coli* (from bloody stool as well as stool from children under five), and three other bacteria genera namely: *Vibrio*, *Salmonella*, and *Shigella*. There were 5.2% (40/772) *Salmonella* spp., 2.2% (17/772) *Shigella* spp., 3.6% (28/772) *Vibrio* sp., and 10.6% (82/772) *E. coli*, from bloody or infant stool (infant <5 years; Table 2). There was however no significant difference (*p* > 0.05) between the different bacteria etiology of diarrhea isolated from all five health facilities (Table 3).

**Table 2:** Bacteria isolates and serotypes.

Variable	Number (%)
*Vibrio* species	*N* = 28
*Vibrio parahaemolyticus*	13 (46.4)
*Vibrio cholerae*	9 (32.1)
*Vibrio fluvialis*	2 (7.1)
*Vibrio* sp. *group*	2 (7.1)
*Vibrio albensis*	1 (3.6)
*Vibrio alginolyticus*	1 (3.6)
*Escherichia coli* serogroups	*N* = 82
O127a	1 (1.2)
O8	4 (4.9)
O159	1 (1.2)
O153	3 (3.6)
O25	1 (1.2)
O142	1 (1.2)
O11	2 (2.4)
O169	1 (1.2)
O18	2 (2.4)
O44	1 (1.2)
O164	1 (1.2)
O6	2 (2.4)
O26	1 (1.2)
O86a	2 (2.4)
O20	1 (1.2)
Non-reactive	58 (70.7)
*Shigella* serogroups	*N* = 17
*Shigella flexneri* type I	2 (11.8)
*Shigella flexneri* type II	7 (5.9)
*Shigella flexneri* type III	2 (11.8)
*Shigella dysenteriae* type II	1 (5.9)
*Shigella dysenteriae* type IV	1 (5.9)
*Shigella boydii* type 12	1 (5.9)
*Shigella sonnei* Phase I	2 (11.8)
Non-reactive	1 (5.9)
*Salmonella* serogroups	*N* = 40
O3, 10	3 (7.5)
O35	1 (2.5)
O4	7 (17.5)
O7	1 (2.5)
O8	1 (2.5)
O9	5 (12.5)
O13	6 (15.0)
Non-reactive	16 (40.0)

**Table 3:** Frequency of bacteria isolated according to health facilities.

Health facility	Frequency of organism				*p*-Value
	*E. coli* (%)	*Salmonella* spp. (%)	*Shigella* spp. (%)	*Vibrio* spp. (%)	
Ga West Municipal Hospital (*N* = 78)	35 (44.9)	23 (29.5)	6 (7.7)	14 (17.9)	0.473
Mayera Health Center (*N* = 31)	19 (61.3)	7 (22.6)	3 (9.7)	2 (6.4)	
Oduman Health Center (N = 34)	18 (52.9)	4 (11.8)	3 (8.8)	9 (26.5)	
Kotoku Health Center (*N* = 17)	8 (47.1)	4 (23.5)	3 (17.6)	2 (11.8)	
St. John’s Hospital (*N* = 7)	3 (42.8)	2 (28.6)	1 (14.3)	1 (14.3)	
Total (*N* = 167)	82	40	17	28	

Other species of bacteria including but not limited to *Klebsiella* (*N* = 50), *Raoultella* (*N* = 16), and *Enterobacter* (*N* = 6) were isolated but not further analyzed for this work.

The *Vibrio* spp. (*N* = 28) were further characterized into six species namely: *Vibrio parahaemolyticus* (13/28, 46.4%), *V. cholerae* (9/28, 32.1%), *Vibrio fluvialis* (2/28, 7.1%), *Vibrio* sp. group (2/28, 7.1%), *Vibrio albensis* (1/28, 3.6%), and *Vibrio alginolyticus* (1/28, 3.6%). Sixteen out of the 17 (94.1%) *Shigella* species were identified as *Shigella flexneri* (11, 64.7%) *Shigella dysenteriae* (2, 11.8%), *Shigella sonnei* (2,11.8%), and *Shigella boydii* (1, 5.9%).

Upon serological analysis, *E. coli* strains were grouped, with the most prominent being serogroups O8 (4, 4.9%) and O153 (3, 3.7%), known to be enterotoxigenic (Escribano et al., 1987; Table 2). Among *Salmonella* isolates, serogroups O13 (15.0%) and O4 (17.5%) were the most dominant. More than one diarrhoeagenic bacteria were isolated from some of the patients; *Salmonella* and *E. coli* (4/792, 0.5%), *Shigella,* and *E. coli* (3/792, 0.4%) were isolated from mainly bloody stools.

### Antimicrobial Susceptibility Profiles of Isolates

**Figure 3:**
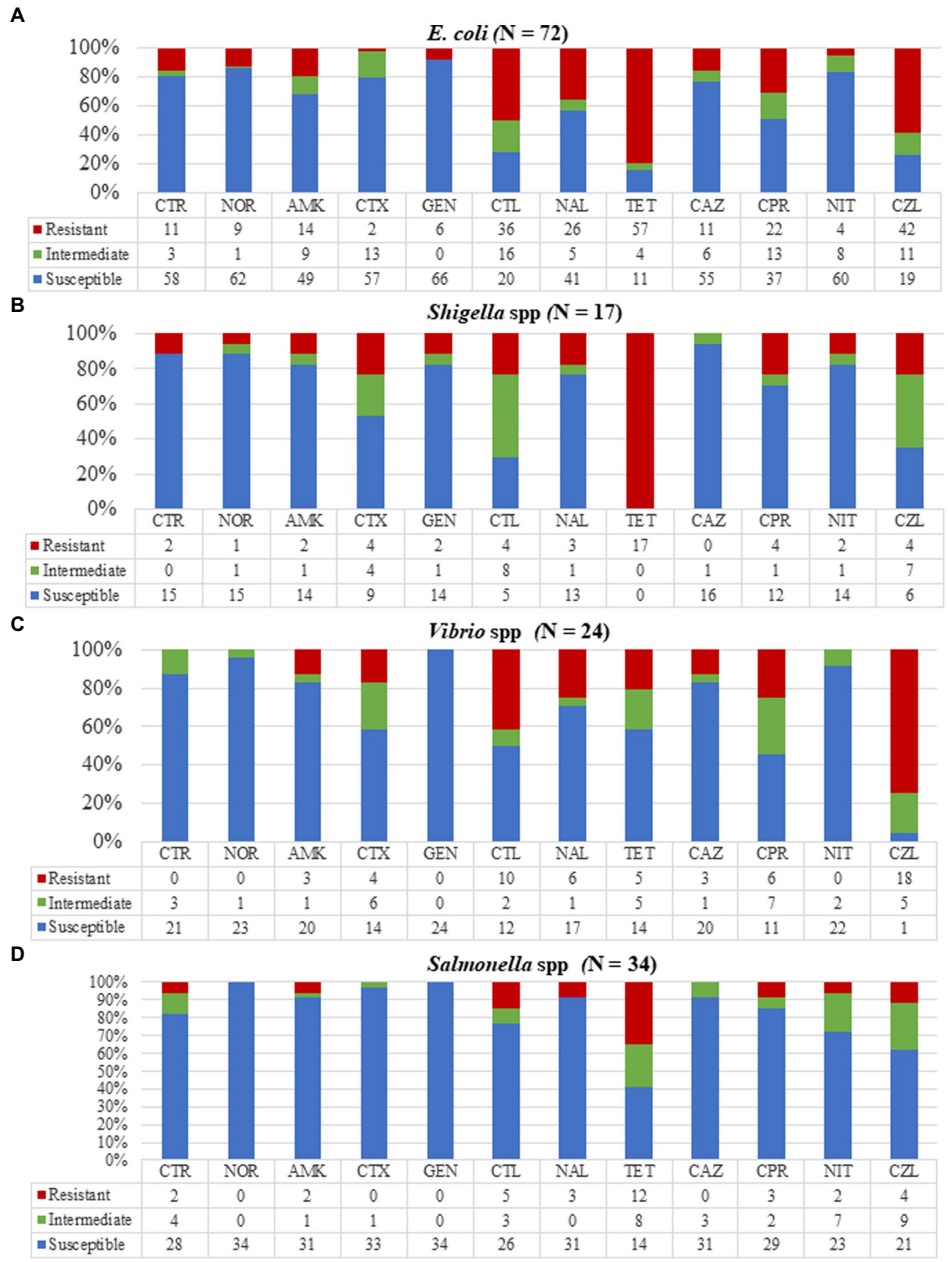
Resistance patterns of the various bacterial strains to 12 antibiotics. **(A)** *Escherichia coli* showing high resistance to tetracycline, cephalothin, and cefazolin. **(B)** *Shigella* isolates were totally resistant to tetracycline with no resistance to ceftazidime. **(C)** *Vibrio* isolates showed high resistance to cefazolin and complete susceptibility to gentamycin. **(D)** All *Salmonella* isolates were susceptible to norfloxacin, cefotaxime, and gentamycin with 35% of isolates resistant to tetracycline. CTR, ceftriaxone; NOR, norfloxacin; AMK, amikacin; CTX, cefotaxime; GEN, gentamycin; CTL, cephalothin; NAL, nalidixic acid; TET, tetracycline; CAZ, ceftazidime; CPR, ciprofloxacin; NIT, nitrofurantoin; and CZL, cefazolin.

Antimicrobial susceptibility testing (AST) was done for 24 out of the 28 isolated *Vibrio* spp. (Figure 3) of which all were susceptible to gentamicin, an aminoglycoside but moderately susceptible to amikacin (14/24, 58.3%). The *Vibrio* isolates showed resistance to cefazolin (18/24, 75%) among which all 13 *V. parahaemolyticus* isolates were resistant to cefazolin. Our *Vibrio* isolates showed 25% (6/24) resistance to ciprofloxacin, used to treat non-cholera *Vibrio* infections. Isolates tested against cefotaxime (21/24, 87.5%) and ceftriaxone (20/24, 83.3%) also used in *Vibrio* infection treatment were highly susceptible. A total of four multi-drug resistant *Vibrio* strains were identified with resistances to fluoroquinolones, cephalosporins, and aminoglycosides.

Out of the 40 *Salmonella* isolates, AST was done for 34 isolates (Figure 3). We detected high resistance of *Salmonella* isolates to tetracycline (12/34, 35.3%) but relatively low resistance to ciprofloxacin (3/34, 8.8%) and ceftriaxone (2/34, 5.9%). The *Salmonella* isolates showed total susceptibility when tested against gentamycin and norfloxacin with no multi-drug resistant *Salmonella* strains however found.

All 17 *Shigella* isolates were tested against our selected antimicrobials (Figure 3) and were found to be resistant to tetracycline. Three multidrug-resistant *Shigella* isolates were identified. One (5.9%) isolate was resistant to three drugs (norfloxacin, ciprofloxacin, and tetracycline), and two (11.8%) were resistant to both norfloxacin and ciprofloxacin.

Antimicrobial susceptibility testing was done on 72/82 *E. coli* isolates and when tested against all our selected antimicrobials, at least one antimicrobial resistance for each isolate was observed (Figure 3). *Escherichia coli* isolates showed the highest resistance to tetracycline (57/72, 79.1%) and cefazolin (43/72, 58.3%). The isolates were highly susceptible to aminoglycosides, amikacin (57/72, 79.2%), and gentamicin (66/72, 91.7%), and nitrofurantoin (60/72, 83.3%) which is prescribed for treating *E. coli* infections. We detected high resistance to one fluoroquinolone, ciprofloxacin (22/72, 30.6%) with two extended-spectrum beta-lactamases-producing *E. coli* identified.

## Discussion

Our routine surveillance study of diarrhea from Ga West Municipality in Ghana from 2017 to 2021 has revealed several important findings. The diversity and frequency of bacteria isolated from this study concur with similar reports from China, Kenya, and India (Mbuthia et al., 2018; William et al., 2020; Wang et al., 2021). The prevalence of *Shigella*, *Salmonella*, *Vibrio*, and diarrheagenic *E. coli* is consistent with previous studies in Ghana and other African countries (Akuffo et al., 2017; Kalule et al., 2019). However, reports from Lompo et al. (2021) did not isolate *Salmonella* from diarrheic stool samples. In our study, we isolated diarrheagenic bacteria from 21% (167/779) of stool samples received which is markedly high compared to previous studies in Ghana and elsewhere (4%; Akuffo et al., 2017; William et al., 2020). This high prevalence may be due to our sampling site, Ga West an area without potable drinking water and thus unsanitary living conditions. As previously reported, diarrheagenic bacteria have been isolated from street food samples in Ghana, which may result in more bacteria diarrhea cases in areas with unsanitary living conditions (Mensah et al., 2002). In a country where most diarrheal cases are thought to only be caused by *V. cholerae*, this revelation is very important. The frequency of *Shigella*, *Salmonella*, *Vibrio*, and enterotoxigenic *E. coli* emphasizes the need to scale up laboratory-based surveillance and appropriate determination of disease etiology for effective treatment of diarrheal diseases. The observable emerging resistance to potent drugs such as ciprofloxacin, norfloxacin, and nalidixic acid used in treating *Vibrio* and *Shigella* is of critical concern.

We observed an emerging resistance of *Vibrio* isolates to ciprofloxacin, a potent cholera medication with 23.1% resistance and 30.8% intermediate resistance consistent with reports in Nigeria, Cameroon, and China (Quilici et al., 2010; de Melo et al., 2011). Our study also identified eight cases of *V. cholerae* which is a public health emergency when not properly managed. In our study, we isolated 13 *V. parahaemolyticus* from diarrhea stools. Two patients infected with *V. parahaemolyticus* presented bloody stool samples with 76.9% (10/13) of *V. parahaemolyticus* infected patients on admission. Bloody stool samples can be a result of *V. parahaemolyticus* damaging the epithelial layer of the small intestines through the creation of cavities (Wang et al., 2022). Classical *V. parahaemolyticus* infection symptoms such as high fever (53.8%), vomiting (53.8%), muscular cramps (46.2%), and general weakness (76.9%) were observed in all infected patients consistent with reports by Jung (2018).

*Vibrio parahaemolyticus* is highly prevalent in marine coastal areas and associated with seafood and endemic in South-Eastern Asia countries including Taiwan, Japan, and China causing many foodborne illnesses (Junhe et al., 2018; Zhao et al., 2020). These *V. parahaemolyticus* were isolated from stools in an area highly deprived of potable water hence depending on the river Densu for water. Residents living along the coast defecate, feed animals, wash and drink from this river, which is of huge public health concern. *Vibrio parahaemolyticus* is known to cause acute gastroenteritis characterized by diarrhea. Although self-limiting, strains of *V. parahaemolyticus* are virulent enough to cause outbreaks and can be fatal (Jung, 2018). With recent reports of antimicrobial-resistant strains (Tan et al., 2020; Jingjit et al., 2021), there is an urgent need to tackle it.

We found *Shigella* isolates to be totally (100%) resistant to tetracycline. This might be a result of the extensive use of tetracycline in Ghana over the years with several reports on antimicrobial resistance to these drugs (Newman et al., 2011; Yevutsey et al., 2017). There might also be some contribution from the irresponsible use in the animal farming industry as a result of easy access to the drug from pharmacies (Wilcox, 2009; Andoh et al., 2016). The *Shigella* spp. were additionally resistant to amikacin (4/17, 23.5%), ciprofloxacin (4/17, 23.5%), nalidixic acid (3/17, 17.6%), and norfloxacin (1/17, 5.9%) with the latter two formerly potent drugs used in *Shigella* dysentery infections in adults (Basnet et al., 2021). Observations of high susceptibility of *Shigella* sp. to ceftriaxone and high resistance to nalidixic acid, tetracycline, and amikacin is consistent with Pourakbari et al. (2010) and Basnet et al. (2021). Parenteral ceftriaxone is highly effective and recommended in the treatment of hospitalized children with severe shigellosis (Ashkenazi et al., 2003) so the emergence of high resistance to this drug is of great concern.

Serological analysis of isolated *E. coli* yielded a high prevalence of serotypes O8 (4.9%) and O153 (3.7%). The serotype O153, an enterotoxigenic *Escherichia coli* (ETEC) has been reported to harbor a large number of virulent genes capable of causing mild to severe infections (Díaz-Jiménez et al., 2020). There are reports of this *E. coli* strain being isolated from patients during an outbreak which may be indicative of its ability to cause an epidemic (Kennedy et al., 2018). Extended-spectrum beta-lactamase (ESBL)-producing *E. coli* were also detected in our study buttressing the high level of resistance occurring in our communities. The transmission and spread of these virulent *E. coli* strains in local communities can be of great danger to public health.

The identification of multi-drug resistant *Vibrio* species in this study continues to show the rising incidence of antimicrobial resistance in the communities. A major problem in Ghanaian communities is the indiscriminate use of antibiotics in animal husbandry and farming sectors as growth supplements for the prevention of infection and subsequently, increasing yield. This practice pre-exposes drugs to these pathogens hence hastening the rate at which antimicrobial resistance develops (Wilcox, 2009; Andoh et al., 2016). Multi-drug resistant *Vibrio* isolates may increase disease severity, morbidity, and mortality and increase constraints on our public health system.

Good hygiene practices will curb the spread of these pathogens since most of these infections are spread through person-to-person contact. The sentinel site chosen in this surveillance study is a community where inhabitants have limited access to pipe-borne water. Residents in some of the communities depend on streams, boreholes, and even rainwater as sources of water for drinking and household chores. Improper disposal of human waste is also another problem in the community. Open defecation into water bodies and bushes coupled with potable water scarcity could account for the high diarrhea cases (Larbi et al., 2021). Practices such as handwashing, proper cooking of food, as well as proper disposal of human and household waste, are known to curb the spread of bacterial pathogens causing diarrhea. Communities must be engaged regularly in the implementation of water, sanitation, and hygiene (WASH) practices. We identified a couple of limitations in the study. Molecular methods such as PCR would have been more sensitive and given a better understanding of the diarrheagenic *E. coli* and ESBLs isolated as compared to serological analysis only.

We did not have data on the total number of individuals presenting diarrhea to the health facilities as samples taken were from only patients who agreed to be in this study.

## Conclusion

This study has shown the diverse bacteria etiology (*Salmonella*, *Shigella*, *Vibrio*, and some *E. coli*) implicated in diarrhea disease and the need for proper differential diagnosis for better treatment outcomes. Resistance observed in all isolates is of public health concern since drugs used in the treatment of Shigellosis, Salmonellosis, and other diarrhea diseases showed marked resistance. This can lead to pressure on our public health system should drugs not work against intended pathogens. A diarrheagenic prevalence rate of 21% further stresses the need for differential diagnosis in diarrhea cases for better treatment outcomes. The presence of multi-drug resistant *Vibrio* isolates and an increase in *Vibrio parahaemolyticus* which is highly associated with food poisoning as well as the high resistant rates of *Shigella* isolates and ESBLs detected is forewarning and raises the need for the implementation of preventive strategies to minimize transmission. Routine antimicrobial susceptibility testing, effective monitoring, and nationwide surveillance of AMR pathogens should be implemented to curb the increase of antimicrobial resistance in Ghana.

## Data Availability Statement

The original contributions presented in the study are included in the article/supplementary material, further inquiries can be directed to the corresponding author.

## Ethics Statement

The studies involving human participants were reviewed and approved by the Institutional Review Board (IRB) of Noguchi Memorial Institute for Medical Research (NMIMR; approval number: 096/16-1; dated on May 3, 2017). It was exempted from additional approval by the Ghana Health Service Ethics Committee which considered the procedures as compliant with routine service for patients care and surveillance. Written informed consent to participate in this study was provided by the participants’ legal guardian/next of kin.

## Author Contributions

DY-M, KK, HK, KT, FA-B, AA-P, and GM: conceptualization, fund acquisition, and writing—review and editing. TA, DA, and AM: investigation. TA, DY-M, and PA: writing—original draft and formal analysis. DY-M, AA-P, GM, TA, DA, and AM: methodology. DY-M: resources, project administration, and supervision. DO, NG, TA, DA, KI, TMa, and TMi: writing—review and editing. AA: project administration. All authors contributed to the article and approved the submitted version.

## Funding

This study was supported by AMED-JICA [the Science and Technology Research Partnership for Sustainable Development (SATREPS); 19jm0110012].

## Conflict of Interest

The authors declare that the research was conducted in the absence of any commercial or financial relationships that could be construed as a potential conflict of interest.

## Publisher’s Note

All claims expressed in this article are solely those of the authors and do not necessarily represent those of their affiliated organizations, or those of the publisher, the editors and the reviewers. Any product that may be evaluated in this article, or claim that may be made by its manufacturer, is not guaranteed or endorsed by the publisher.
